# Is there a difference in prevalence of helminths between households using ecological sanitation and those using traditional pit latrines? A latrine based cross sectional comparative study in Malawi

**DOI:** 10.1186/s13104-017-2519-7

**Published:** 2017-06-09

**Authors:** Save Kumwenda, Chisomo Msefula, Wilfred Kadewa, Yohane Diness, Charles Kato, Tracy Morse, Bagrey Ngwira

**Affiliations:** 1 College of Medicine, University of Malawi, Chichiri, P/Bag 360, Blantyre 3, Malawi; 2 The Polytechnic, University of Malawi, Chichiri, P/Bag 303, Blantyre 3, Malawi; 3 Lilongwe University of Agriculture and Natural Resources, P. O. Box 219, Lilongwe, Malawi; 4 School of Bio-security, Biotechnical & Laboratory Sciences, College of Veterinary Medicine, Animal Resources & Bio-security, Makerere University, Kampala, Uganda; 5 Department of Civil and Environmental Engineering, University of Strathclyde, Glasgow, UK

**Keywords:** Pit latrines, EcoSan, Soil transmitted helminths, *Ascaris lumbricoides*, Human faecal matter and risk

## Abstract

**Background:**

Studies have shown that households using sludge from human excreta for agriculture are at an increased risk of soil transmitted helminths. However, while use of ecological sanitation (EcoSan) latrines is increasing in most African countries including Malawi, few studies have been done to check whether use of such sludge could potentially increase the prevalence of helminthic infections among household members as a results of exposure to faecal sludge/compared to use of traditional latrines.

**Methods:**

A cross sectional study was done targeting households using EcoSan and traditional pit latrines. Samples were collected from both types of latrines in Chikwawa (rural) and Blantyre (urban) districts. These two districts have a high number of EcoSan latrines in southern region of Malawi. 156 latrines were sampled (n = 95 traditional; n = 61 EcoSan), and processed following standard guidelines using modified triple floatation method. Identification of helminth ova (*Ascaris lumbricoides*, hookworms, *Trichuris trichiura*, *Taenia* spp. and *Diphyllobothrium latum*) was done using standard microscopy methods. The difference between the prevalence and mean concentration of helminths between the two types of latrines was tested using Chi Square and t test respectively.

**Results:**

Of the total latrines tested, 85.9% (n = 134) had at least one species of helminth while 84.6% (n = 132) had at least a STH, with 82.0% (n = 50) in EcoSan and 86.3% (n = 82) in traditional pit latrines. There was no significant difference between the prevalence of helminths in EcoSan and traditional pit latrines [χ^2^ = 0.43 (1), P = 0.5]. The prevalence of *Ascaris lumbricoides* was significantly higher in EcoSan than in traditional pit latrines [χ^2^ = 5.44 (1) p = 0.02] while prevalence of hookworms was significantly higher in traditional pit latrines than in EcoSan latrines [χ^2^ = 13.98 (1) p < 0.001]. The highest concentration of helminths per gram of faecal sludge was in traditional pit latrines [31.2 (95% CI 19.1–43.2)] than in EcoSan latrines [26.4 (95% CI 16.5–36.3)].

**Conclusion:**

There was no significant difference between overall prevalence of helminths between households using EcoSan and those using traditional pit latrines. However, *Ascaris lumbricoides* was significantly higher in households using EcoSan latrines. EcoSan users need awareness on safe ways of handling faecal sludge in order to reduce chances of reinfection from *Ascaris lumbricoides*. Further research should be undertaken on household members to identify those infected and potential routes of infection to enable preventive targeting.

## Background

Soil transmitted helminths (STH) are the most common infectious agents of humans in developing countries [1]. STHs affect 24% of the world’s population [2], and are most prevalent in tropics and sub-tropical regions where their existence has been traced to ancient times through sediment analysis [3, 4]. In 2013, *Ascaris* affected an estimated 807–1221 million people in the world mostly in developing countries [5–7], with an average prevalence of 21.2, 12.2 and 33.1% for hookworms, *Ascaris lumbricoides* and *Trichuris trichiura* respectively reported in southern sub Saharan Africa [8]. In Malawi, few studies have been done to estimate the household prevalence of STHs, however a prevalence of 0.4, 1.3 and 0.5% for *Schistosoma mansoni*, hookworms and *Ascaris lumbricoides* respectively was reported in primary school children, with a higher prevalence in urban (16.5%) than rural children (3.6%) [9]. The health effects of STHs are well known, and although some STHs alone do not cause severe disease, when combined with other diseases, they may lead to complications [10], including increased susceptibility to malaria, malnutrition and anaemia [11]. With specific reference to pregnancy, infection with *Ascaris lumbricoides* was reported to make women more susceptible to earlier first births and shortened inter-birth intervals, while hookworm was associated with delayed first pregnancy [12, 13].

The prevalence of STHs remains high despite mass drug administration (MDA) in at risk countries, which emphasizes the need to reduce STH prevalence through both MDA and reduced environmental exposure [14]. Risk factors for STHs have been reported to include poverty, use of unimproved water sources, use of unimproved sanitation, not wearing shoes, poor education, eating raw vegetables, which are not properly washed, and unhygienic practices [15–19].

In Malawi, the Government promotes use of improved sanitation in order to reduce diarrhoeal and STH infections. This includes upgrading traditional pit latrines with a slab, flush toilets, ventilated improved pit latrines and the ecological sanitation (EcoSan) latrine. Current coverage of household pit latrines is 79.9 and 78.6% in urban and rural areas of Malawi respectively [20]. The traditional pit latrines comprise all the pit latrines whether improved or not, ventilated improved pit latrines but excludes EcoSan [21, 22].

EcoSan latrines are a sanitation option aimed at closing the plant nutrient loop by making the nutrients in human faecal matter available to plants again. After defaecating in an EcoSan latrine, two cups of soil and one cup of ash are added to reduce smell and assist in treating the sludge. After using the latrine for defecation for 6 months or more, the latrine pit or vault is sealed for another 6 months to allow the pathogens to be killed. Thereafter, the contents are removed and used as soil conditioner as well as fertilizer for crops [23, 24]. Harvested sludge is often used for growing crops and vegetables in the fields, and consequently, producing safe sludge free of STHs, especially *Ascaris lumbricoides,* is extremely important [25]. It has been reported that EcoSan latrines do not completely inactivate STH eggs in the sludge while in the vault/pit, due to inadequate environmental conditions including ambient temperatures during digestion [26, 27]. High lethal temperatures of >55 °C are required to inactivate *Ascaris lumbricoides* and as such it is used as an indicator organism for faecal sludge safety due to its resistance to environmental conditions, and its 100% risk for exposed population [28, 29].

In Malawi, EcoSan latrines were introduced in 2001 as a sanitation option by Non-Governmental Organizations (NGOs) and currently account for approximately 0.3% of toilets [20].

The reuse of faecal sludge from EcoSan latrines has become a public health concern because it puts users and the general population at risk of STH infections through direct contact with sludge during harvesting, storage, transportation to field, application in the fields and through environmental pollution, particularly due to the lack of protective wear (e.g. gloves, masks, boots) [30, 31]. In Malawi, EcoSan products are primarily used in personal agricultural fields, and this potentially puts them at an increased risk of STH infections [32]. Studies in South Africa and Malawi, showed the main concern with using EcoSan sludge was the pathogen content [25, 33]. This concern was compounded by the practices of EcoSan latrines owners, where they were found to store their harvested sludge behind their households on bare ground. This led to uncontrolled spreading of manure around the household environment increasing the chances of infection [33]. Alternatively, the main concern for traditional pit latrines is ground water contamination, with no direct link to STH transmission made by users due to lack of direct contact with faecal sludge, therefore they are not seen as a potential route for STH infection [34]. As such, this study aimed to determine if the use of EcoSan was related to a higher prevalence of STHs in household members. This was achieved by comparing STH presence in EcoSan and traditional latrines respectively based on the modified triple floatation protocol which gives a representative estimation of helminths prevalence among household members from deposited faecal matter [35].

## Methods

### Study design and study area

A comparative cross sectional study was undertaken from September 2015 to January, 2016 and targeted households using EcoSan and traditional pit latrines in the two districts of Blantyre and Chikwawa in Southern Region of Malawi (Table 1).

**Table 1:** Description of study areas

Characteristic	Blantyre (urban and rural)	Chikwawa (rural)
Area, km^2^ [36]	2, 012	4, 755
Population [36]	1, 239, 648	518, 287
Elevation, m [37]	1, 001–1, 500	51–100
Temperature, °C (average: min–max) [38]	9.9–39.5	10–45.6
Rainfall, mm (average: min–max) [38]	0–142.7	0–86.4

The districts were chosen to represent the two extremes of temperature in Malawi which may impact on STH survival. Four specific sample areas in each District were randomly selected based on the presence of EcoSan latrines using data from both Government and NGOs [39]. EcoSan latrines were selected from peri-urban areas of Blantyre (Chemusa, Angelo Govea, Chilomoni) and in Blantyre rural (Lirangwe). In Chikwawa, they were selected from the rural villages of Kaputeni, Ng’ombe, Zimola and Tomali. Traditional latrines were outside a 500 metres radius from EcoSan latrines to reduce the risk of contamination from EcoSan faecal sludge in the environment.

### Sample size and sample collection

The required sample size was based on the estimated the proportion of EcoSan latrines with at least a helminth which was 86% in rural KwaZulu-Natal in South Africa [35]. Considering the practices on storage, transportation and use of sludge from EcoSan latrines, we estimated a lower proportion of ordinary pit latrines to have Ascaris ova in their sludge. This was estimated at 56%. Using “*sampsi”* command in Stata 12, a sample size of 53 EcoSan and 53 ordinary pit latrines was required at α of 0.05 and power of 0.9. After considerations of non-responses, geographical location representation and number of EcoSan and ordinary pit latrines available in the study areas a final sample size of 61 EcoSan latrines and 95 ordinary pit latrines was decided. The total sample size was 156 latrines which were selected in different locations in Blantyre and Chikwawa Districts as shown in Table 2.

**Table 2:** Number of latrines sampled from each location

District	Location/village	EcoSan latrines	Traditional latrines	Total
Blantyre	Chemusa	13	0	13
	Angelo Govea	13	0	13
	Chilomoni	11	20	31
	Lirangwe	2	0	2
Chikwawa	Ng’ombe	14	0	14
	Zimola	4	0	4
	Kaputeni	4	60	64
	Tomali	0	15	15
Total		61	95	156

Ninety-five (95) traditional (peri-urban = 20; rural = 75) and 61 EcoSan (peri-urban = 37; rural = 24) latrines were sampled in total. Data were collected from each household as follows:

### Demographic data

A short household demographic questionnaire was administered to the household heads before sampling the latrine.

### Faecal sludge

Sampling sought to determine the concentration of STHs in faecal sludge from latrines in current use as an estimation of household burden. The sample was collected from the top 30 cm of the faecal sludge pit after thorough mixing with a stick. The sample was collected by scooping at the centre and sides of the pit using a locally fabricated adjustable scooper. Three scoops were made per latrine with a total weight ranging from 100 to 300 g. The samples were put in labelled plastic bottles and put in a cooler box for transportation to the College of Medicine laboratory within 3 h of collection. The samples were kept in a refrigerator and processed within 2 days. Recovery of helminths from the latrine faecal matter was done using the modified triple floatation protocol [40]. An Olympus BX41 microscope was used to identify and enumerate the eggs. The modified triple floatation protocol has been reported to recover 77% of the helminths from the sample and this was significantly better than any other tested method [41].

### Data analysis

Demographic and laboratory data were entered in Excel and then imported into Stata 12 for analysis. Chi square test and t test were used to check the significance of the differences between overall prevalence and intensities of helminths between the two types of latrines. Differences at p < 0.05 were deemed to be significant.

### Ethical considerations

A written informed consent form translated in local language (Chichewa) was administered and signed by the household head where a latrine was selected for inclusion in the study. An informed verbal consent was also obtained during a short questionnaire administration by the household member who took part in answering the questions. Each latrine enrolled assigned a unique identifying number and all the records were kept in a separate folder for each latrine. Names of households were kept anonymous and confidential. Participants were given the opportunity to ask questions and had chance refuse being enrolled and also to drop-out anytime during the study. Study participants were not exposed to any risks as they were only involved in answering questions from the interview and observe the sampling process. No samples from people were collected. Ethical approval was obtained from the University of Malawi, College of Medicine Research Ethics Committee in October 2014 (P.04/14/1565).

## Results

### Demographic characteristics of respondents

Higher average income and formal education were both significant factors in the ownership and use of EcoSan Latrines while marital status and sex were not significant as shown in Table 3.

**Table 3:** Demographic characteristics of respondents

Characteristic	Category	EcoSan latrines	Traditional pit latrines	p value
Sex	Male	21 (34.4%)	37 (38.9%)	0.57
	Female	40 (65.6%)	58 (61.1%)	
Marital status	Married	50 (82.0%)	70 (73.7%)	0.43
	Single	4 (6.6%)	8 (8.5%)	
	Divorced	4 (6.6%)	7 (7.4%)	
	Widowed	3 (4.9%)	10 (10.5%)	
Average household income per month	<$7.00	3 (4.9%)	13 (13.7%)	0.001*
	$7–$13.00	5 (8.2%)	23 (24.2%)	
	>$13.00–$27.00	9 (14.8%)	24 (25.3%)	
	>$27.00	44 (72.1%)	35 (36.8%)	
Education	No formal education	2 (3.3%)	15 (15.8%)	0.004*
	Primary	23 (37.7%)	47 (49.5%)	
	Secondary	31 (50.8%)	24 (25.3%)	
	Tertiary	5 (8.2%)	9 (9.5%)	

* Presence of a significant relationship using Chi square test

### Prevalence of helminths in latrines

The total numbers of STH and other helminths that were found in the samples in form of eggs were counted for each household latrine. Table 4 indicates the prevalence found by latrine type.

**Table 4:** Prevalence of helminthes by latrine type

Name of helminth	EcoSan latrines	Traditional pit latrines	Total	P value
STH				
*Ascaris lumbricoides*	38 (62.3%)	41 (43.2%)	79 (50.6%)	0.02*
Hookworms	34 (55.7%)	79 (83.2%)	113 (72.4%)	0.001*
*Trichuris trichiura*	2 (3.3%)	1 (1.1%)	3 (1.9%)	0.323
*Taenia* spp.	20 (32.8%)	43 (45.3%)	63 (40.4%)	0.121
Other helminths				
*Schistosoma mansoni*	3 (5.0%)	15 (15.8%)	18 (11.5%)	0.038*
*D. latum*	1 (1.6%)	5 (5.3%)	6 (3.8%)	0.251
At least one helminth	51 (83.6%)	83 (87.4%)	134 (85.9%)	0.051

* Presence of a significant relationship using Chi Square test

We found that 85.9% (n = 134) of the total latrines tested had at least one species of helminth while 84.6% (n = 132) had at least a STH with 82.0% (n = 50) in EcoSan and 86.3% (n = 82) in traditional pit latrines. There was no significant difference between the prevalence of helminths in EcoSan and traditional pit latrines [χ^2^ = 0.43 (1), P = 0.5]. The prevalence of *Ascaris lumbricoides* was significantly higher in EcoSan than in traditional pit latrines [χ^2^ = 5.44 (1) p = 0.02] while that of hookworms was significantly higher in traditional pit latrines than in EcoSan latrines [χ^2^ = 13.98 (1) p < 0.001]. The prevalence of other STH was similar in both types of latrines. *Schistosoma mansoni*, though not soil transmitted, was significantly higher in traditional pit than in EcoSan latrines. We also found a fish helminth, *Diphyllobothrium latum (D. latum),* which has never been reported before in Malawi (Table 4). In terms of location, the results also showed no significant difference [χ^2^ = 0.0003 (1), P = 0.9] between prevalence of at least a helminth in latrines in peri-urban areas of Blantyre (86.4%, n = 51) and those in rural areas of Chikwawa (85.6%, n = 83).

### Average concentration of helminth ova per gram of faecal sludge

In order to know the concentration of helminths per gram of faecal sludge by latrine type, mean values in helminths per gram were calculated (Table 5).

**Table 5:** Mean concentration of helminths measured in helminths per gram (95% CI) by latrine type

Name of helminth	EcoSan latrines	Traditional pit latrines	Total	P value*
STH				
*Ascaris lumbricoides*	3.3 (2.0–4.5)	4.7 (1.8–7.6)	4.1 (2.3–5.9)	0.43
Hookworms	19.6 (11.4–27.9)	21.1 (12.8–29.4)	20.5 (14.6–26.5)	0.80
*Trichuris trichiura*	0.1 (0–0.3)	0.02 (0.0–0.06)	0.06 (0.02–0.14)	0.17
*Taenia* spp.	3.0 (1.2–4.9)	4.7 (0.8–8.6)	4.0 (1.6–6.5)	0.52
Other helminths				
*Schistosoma mansoni*	0.2 (0.0–0.4)	0.6 (0.2–0.9)	0.4 (0.2–0.6)	0.11
*D. latum*	0.2 (0–0.5)	0.1 (0.0–0.2)	0.1 (0.0–0.3)	0.25
Overall	26.4 (16.5–36.3)	31.2 (19.1–43.2)	29.3 (21.1–37.6)	0.57

* P value for the difference in means between EcoSan and traditional pit latrines using t test

Traditional pit latrines have high mean helminthic levels though the difference is not statistically significant (Table 5). Similarly, no significant difference was observed between the mean distributions of helminth eggs when grouped by district (Table 6). The maximum helminths eggs per gram of faecal sludge for EcoSan latrines were 173, 21, 41, 5, 5, 10 while in faecal sludge from traditional pit latrines were 301, 116, 180, 10, 2, 3 for hookworms, *Ascaris lumbricoides, Taenia* spp.*, Schistosoma mansoni, Trichuris trichiura* and *D. latum* respectively.

**Fig. 1:**
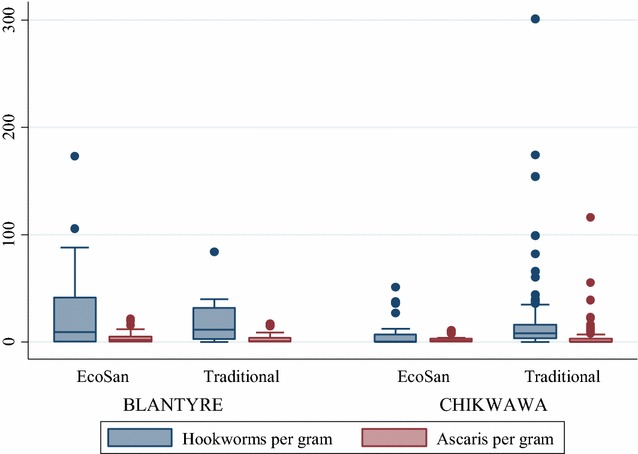
Box plot for concentration of helminths in faecal sludge. The* graph* gives a picture of how the helminths were distributed in traditional pit and EcoSan latrines in Blantyre and Chikwawa

Table 6 shows that in Blantyre, there were more helminths per gram of faecal sludge in EcoSan latrines than in traditional pit latrines while in Chikwawa, more helminths per gram were found in traditional pit latrines though not statistically significant (p > 0.05). There was no statistical significant difference in the concentration of helminths per gram between urban and rural latrines (p > 0.05). Figure 1 gives a picture of how the hookworms and *Ascaris lumbricoides* were distributed in the two types of latrines between Blantyre and Chikwawa.

**Table 6:** Concentration of helminths per gram of faecal sludge in EcoSan and traditional pit latrines by district

Name of helminth	Blantyre District		Chikwawa District		P value*
	EcoSan latrines	Traditional pit latrines	EcoSan	Traditional pit latrines	
STH					
*Ascaris lumbricoides*	3.9	3.3	2.3	5.1	0.46
Hookworms	26.2	19.7	8.0	21.5	0.81
*Trichuris trichiura*	0.1	0	0.2	0.0	0.18
*Taenia* spp.	4.7	2.8	0.1	5.2	0.53
Other helminths					
*Schistosoma mansoni*	0.1	0.1	0.3	0.7	0.11
*D. latum*	0.3	0.2	0	0.1	0.67
Overall (all helminths)	35.1	26.0	11.0	32.6	0.58

* P value for the mean concentration of helminths by district using t test

The median helminths eggs per gram for hookworms was 5.72 and 9.0 for EcoSan and traditional pit latrines respectively while for *Ascaris lumbricoides*, it was 9.0 and 0 in EcoSan and traditional pit latrines respectively (Fig. 1). Table 7 shows the types of helminths present in one latrine sample.

**Table 7:** Total number of helminths types present in a latrine faecal sample

Total number of helminths types present in a latrine faecal sample	EcoSan latrines (%)	Traditional pit latrines (%)	Total number of latrines (% of total)
None	10 (45.5)	12 (54.5)	22 (14.1)
One	22 (45.8)	26 (54.2)	48 (30.8)
Two	13 (34.2)	25 (65.8)	38 (24.4)
Three	13 (37.1)	22 (62.9)	35 (22.4)
Four	3 (27.3)	8 (72.7)	11 (7.1)
Five	0 (0.0)	2 (100.0)	2 (1.3)
Six	0 (0.0)	0 (0.0)	0 (0.0)
Total	61	95	156

It was found that most latrines had at least one type of helminth (30.8%) while none had all the six helminths present (Table 7). It was also found that 40.4% (63) of the latrines had both *Ascaris lumbricoides* and hookworms while only one EcoSan latrine in Chikwawa had all the STH.

## Discussion

The results of this study are from samples collected from latrines used by household members for defecation. These results have been used to estimate the prevalence of helminths among the 156 households that use the latrines. The overall prevalence of helminths was found to be 85.9% (EcoSan = 83.6%; traditional pit latrines = 87.4%) with 84.6% having at least one STH present. This was higher than what was found in a similar study done in South Africa in 2010. The study found the prevalence of at least a STH (*Ascaris lumbricoides, Taenia* spp., *Trichuris trichiura*) in a family toilet of 73%. In terms of specific STH, our study results were similar to the South African study only in terms of *Ascaris lumbricoides* prevalence (50.6% versus 59%) while *Trichuris trichiura* and *Taenia* spp. were very low in the current study [35]. Our overall prevalence was due mainly to high levels of hookworms, which were not considered in the South African study [35]. In terms of the differences between overall prevalence of helminths between EcoSan and traditional pit latrines, we found that there was no significant difference between the two types of latrines. In addition, there were no significant differences between prevalence of STH in urban (Blantyre) and rural (Chikwawa) latrines and this was attributed to overall high transmission rates. With high prevalence rates it is difficult to observe the differences between latrine types and locations. However, we found that *Ascaris lumbricoides* was significantly higher in EcoSan than in traditional pit latrines while hookworms and *Schistosoma mansoni* were significantly higher in traditional pit latrines. The results were not a surprise considering the high risk among those using EcoSan latrines from improper handling of sludge which leads to reinfection by STHs especially *Ascaris lumbricoides*. According to a study done in Malawi in 2013, sludge (after 6 months storage) from EcoSan was found to contain viable *Ascaris* ova and presented a risk to users [32]. However for hookworms, the use of ash and the dry conditions in EcoSan latrines, may explain the deactivation and therefore lower concentrations in these latrines [42].

Overall, in terms of concentration of helminth eggs per gram of faecal sludge, EcoSan had lower helminths per gram of sludge than traditional pit latrines. In terms of location, Blantyre had high concentration of helminths in sludge from EcoSan latrines than in sludge from traditional pit latrines. In EcoSan latrine sludge, lower levels of helminths is expected because of the practices of making them dry and adding ash. The practices encouraged are those that encourage deactivating of pathogens because the sludge is reused for agriculture purposes. However, due to poor practices that lead to reinfection from environmental resistant helminth, high *Ascaris lumbricoides* concentrations were expected. Ascaris concentration was only high in sludge from EcoSan latrines in Blantyre. The Chi square test showed no statistical significant difference between average concentrations in EcoSan and traditional pit latrines. The high prevalence of helminth in sludge from latrines implies that about one in eight households have a user of latrine who is infected with helminths which means there will be high transmission rates especially if hygienic principles are not followed. The lack of significant differences in concentrations may be due to the high transmission rates anticipated. Despite having prevalence similar to a study in done in South Africa, the concentrations in this study were much lower. The South African study where 120 urine diverting toilets were sampled, found a mean concentration of 33,400 helminths per gram and a median value of 3000 helminths per gram of faecal sludge [35]. Our study had average concentrations of lower than 50 helminths per gram of faecal sludge. The low concentrations could be due to the improvements in sanitation and other water and sanitation projects being implemented by Malawi Government and Non-Governmental organization (NGOs) in the study areas during the time of the survey.

It was found that 14.1% (22) latrines had no helminths present in the faecal sludge sampled from them. Most of these were traditional pit latrines. The highest number of latrines had one types of helminths while none had all the six helminths present. The most common helminth was hookworm followed by *Ascaris lumbricoides,* which is consistent with the findings of other studies [9, 43].

It was also found that those using EcoSan users had more income and were more educated than those using traditional pit latrines. EcoSan latrines were more economically demanding in the short terms than the traditional pit latrines which required less money during construction. This might be the reason household heads with EcoSan had more income than those using traditional pit latrines. For example, an EcoSan (skyloo) needed about $272 to construct in 2015 while a traditional pit latrine in the village required from just energy and local resources to about $136 if built using bought materials and hired labour [44]. The construction cost of EcoSan latrine is in line with the name that people have given to the EcoSan; they called it *“chimbudzi chamakono”* which means a modern latrine. This also aligns with the fact that those using EcoSan were more literate than those using traditional pit latrines as education has been related to economic status and ability to take up new innovations in most studies [45, 46]. It is also argued that having the means to adopt an innovation is one of the important steps in diffusion of innovations theory [47].

### Challenges and limitations of the study

EcoSan latrines have a maximum depth of 1.5 m and this made it easy to reach the contents with a sampler. However, the use of ash and soil made it difficult to mix the contents and get a representative sample. In ordinary traditional pit latrines which were newly built, it was difficult to get representative sample as most of them were more than 3 m deep.

## Conclusion

There was no significant difference between overall prevalence of helminths between households using EcoSan and those using traditional pit latrines. However, *Ascaris lumbricoides* was significantly higher in households using EcoSan than in those using traditional pit latrines while hookworms and *Schistosoma mansoni* were significantly higher in traditional pit latrines. The study stresses the need for awareness among EcoSan users on safe ways of handling faecal sludge in order to reduce chances of reinfection from *Ascaris lumbricoides*. Further research to consider testing the household members in order to identify those infected so that interventions can better target them. More research is required to check if the helminths identified in EcoSan latrines are deactivated after the 6 months treatment period.

## Abbreviations

CARTA: Consortium for Advanced Research Training in Africa
CI: confidence interval
DelPHE: development partnerships in higher education
DfID: Department for International Development
EcoSan: ecological sanitation
Ph.D.: Doctor of Philosophy
SACORE: Southern African Consortium for Research Excellence
SCHI: Scotland Chikwawa Health Initiative
Sida: The Swedish International Development Cooperation Agency
Rpm: revolutions per minute
µm: micrometers
UK: United Kingdom
*D. latum*: *Diphyllobothrium latum*

## References

[1] Hotez PJ, Brindley PJ, Bethony JM, King CH, Pearce EJ, Jacobson J (2008). Helminth infections: the great neglected tropical diseases. J Clin Invest.

[2] WHO. Soil-transmitted helminth infections. WHO. 2015. http://www.who.int/mediacentre/factsheets/fs366/en/. Accessed 29 Aug 2015.

[3] Le Bailly M, Landolt M, Bouchet F (2012). First World War German soldier intestinal worms: an original study of a trench latrine in France. J Parasitol.

[4] Reinhard KJ, Araújo A, Sianto L, Costello JG, Swope K (2008). Chinese liver flukes in latrine sediments from Wong Nim’s property, San Bernardino, California: archaeoparasitology of the Caltrans District Headquarters. J Parasitol.

[5] Centers for disease control. Parasites—Ascariasis. 2013. http://www.cdc.gov/parasites/ascariasis/. Accessed 25 Dec 2015.

[6] Kanneganti K, Makker JS, Remy P (2013). *Ascaris lumbricoides*: to expect the unexpected during a routine colonoscopy. Case Rep Med..

[7] Yamashita ET, Takahashi W, Kuwashima DY, Langoni TR, Costa-Genzini A (2013). Diagnosis of *Ascaris lumbricoides* infection using capsule endoscopy. World J Gastrointest Endosc.

[8] Pullan RL, Smith JL, Jasrasaria R, Brooker SJ (2014). Global numbers of infection and disease burden of soil transmitted helminth infections in 2010. Parasit Vectors.

[9] Bowie C, Purcell B, Shaba B, Makaula P, Perez M (2004). A national survey of the prevalence of schistosomiasis and soil transmitted helminths in Malaŵi. BMC Infect Dis.

[10] Xiao P-L, Zhou Y-B, Chen Y, Yang Y, Shi Y, Gao J-C, et al. Prevalence and risk factors of *Ascaris lumbricoides* (Linnaeus, 1758), *Trichuris trichiura* (Linnaeus, 1771) and HBV infections in Southwestern China: a community-based cross sectional study. Parasit Vectors. 2015. http://www.ncbi.nlm.nih.gov/pmc/articles/PMC4690309/. Accessed 19 Jan 2016. 10.1186/s13071-015-1279-2 PMC4690309 26704345

[11] Degarege A, Veledar E, Degarege D, Erko B, Nacher M, Madhivanan P (2016). Plasmodium falciparum and soil-transmitted helminth co-infections among children in sub-Saharan Africa: a systematic review and meta-analysis. Parasit Vectors..

[12] Blackwell AD, Tamayo MA, Beheim B, Trumble BC, Stieglitz J, Hooper PL (2015). Helminth infection, fecundity, and age of first pregnancy in women. Science.

[13] Shiferaw MB, Mengistu AD (2015). Helminthiasis: hookworm infection remains a public health problem in Dera District, South Gondar, Ethiopia. PLoS ONE.

[14] Steinmann P, Yap P, Utzinger J, Du Z-W, Jiang J-Y, Chen R (2015). Control of soil-transmitted helminthiasis in Yunnan province, People’s Republic of China: experiences and lessons from a 5-year multi-intervention trial. Acta Trop.

[15] Kattula D, Sarkar R, Ajjampur SSR, Minz S, Levecke B, Muliyil J (2014). Prevalence & risk factors for soil transmitted helminth infection among school children in south India. Indian J Med Res.

[16] Menzies SK, Rodriguez A, Chico M, Sandoval C, Broncano N, Guadalupe I (2014). Risk factors for soil-transmitted helminth infections during the first 3 years of life in the tropics; findings from a birth cohort. PLoS Negl Trop Dis..

[17] Huat LB, Mitra AK, Jamil NIN, Dam PC, Mohamed HJJ, Muda WAMW (2012). Prevalence and risk factors of intestinal helminth infection among rural malay children. J Glob Infect Dis.

[18] Matthys B, Bobieva M, Karimova G, Mengliboeva Z, Jean-Richard V, Hoimnazarova M (2011). Prevalence and risk factors of helminths and intestinal protozoa infections among children from primary schools in western Tajikistan. Parasit Vectors.

[19] Phiri KS (2001). The prevalence, intensity and ecological determinants of helminth infection among children in an urban and rural community in southern Malawi. Malawi Med J.

[20] National Statistical Office, ICF International. Malawi Demographic and Health Survey 2015–2016. Zomba, Malawi, and Rockville, Maryland, USA: NSO and ICF International; 2016. http://www.dhsprogram.com/pubs/pdf/PR73/PR73.pdf. Accessed 14 Jun 2016.

[21] Grimason AM, Davison K, Tembo KC, Jabu GC, Jackson MH (2000). Problems associated with the use of pit latrines in Blantyre, Republic of Malawi. J R Soc Promot Health.

[22] Ministry of irrigation and water development. Low cost latrine technologies. Malawi Government. 2011.

[23] Jensen PK, Phuc PD, Konradsen F, Klank LT, Dalsgaard A (2009). Survival of Ascaris eggs and hygienic quality of human excreta in Vietnamese composting latrines. Environ Health.

[24] Mehl JA. Pathogen destruction and aerobic decomposition in composting latrines: a study from rural panama. Michigan Technological University. 2008. http://cee.eng.usf.edu/PeaceCorps/5%20-%20Resources/Theses/Sanitation/2008Mehl.pdf. Accessed 12 May 2014.

[25] Jimenez B, Austin A, Cloete E, Phasha C (2006). Using Ecosan sludge for crop production. Water Sci Technol.

[26] Gajurel DR, Wendland IC. Ecological sanitation and associated hygienic risk. Overv Exist Policy Mak Guidel Res WECF Women Eur Common Future. 2007. http://www.ircwash.org/sites/default/files/Gajurel-2007-Ecological.pdf. Accessed 7 Sept 2016.

[27] Redlinger T, Graham J, Corella-Barud V, Avitia R (2001). Survival of fecal coliforms in dry-composting toilets. Appl Environ Microbiol.

[28] McKinley JW, Parzen RE, Mercado Guzman A (2012). Ammonia inactivation of ascaris ova in ecological compost by using urine and ash. Appl Environ Microbiol.

[29] Schönning C, Stenström TA. Guidelines on the safe use of urine and faeces in ecological sanitation systems. EcoSanRes Programme, Stockholm; 2004.

[30] Lam S, Nguyen-Viet H, Tuyet-Hanh TT, Nguyen-Mai H, Harper S (2015). Evidence for public health risks of wastewater and excreta management practices in Southeast Asia: a scoping review. Int J Environ Res Public Health.

[31] Trang DT, Mølbak K, Cam PD, Dalsgaard A (2007). Helminth infections among people using wastewater and human excreta in peri-urban agriculture and aquaculture in Hanoi, Vietnam. Trop Med Int Health.

[32] Morgan P, Mekonnen A Tesfaye. Paving the Way to Scaling Up Ecosan in Malawi. Share research. 2013. http://www.shareresearch.org/LocalResources/Morgan_and_Mekonnen_2013_Paving_the_Way_to_Scaling_Up_Ecosan.pdf. Accessed 30 Apr 2013.

[33] Kumwenda S, Msefula C, Kadewa W, Ngwira B, Morse T, Ensink JHJ (2016). Knowledge, attitudes and practices on use of Fossa Alternas and double vault urine diverting dry (DVUDD) latrines in Malawi. J Water Sanit Hyg Dev.

[34] Graham JP, Polizzotto ML (2013). Pit latrines and their impacts on groundwater quality: a systematic review. Environ Health Perspect.

[35] Trönnberg L, Hawksworth D, Hansen A, Archer C, Stenström TA (2010). Household-based prevalence of helminths and parasitic protozoa in rural KwaZulu-Natal, South Africa, assessed from faecal vault sampling. Trans R Soc Trop Med Hyg.

[36] GoeHive. GeoHive—Malawi population statistics. 2016. http://www.geohive.com/cntry/malawi.aspx. Accessed 8 Oct 2016.

[37] MASDAP. Elevation Map of Malawi [Internet]. 2013. http://www.masdap.mw/documents/151. 8 Oct 2016.

[38] Department of Climate Change and Meteorological Services (2016). Malawi meteorological data.

[39] MOH (2015). Malawi govt report on sanitation coverage by district.

[40] Moodley P, Archer C, Hawksworth D, Leibach L (2008). South Africa, water research commission. Standard methods for the recovery and enumeration of helminth ova in wastewater, sludge, compost and urine-diversion waste in South Africa: report to the Water Research Commission.

[41] Buckley CA (2008). South Africa, water research commission. Research into UD/VIDP (urine diversion ventilated improved double pit) toilets: physical and health-related characteristics of UD/VIDP vault contents.

[42] Berendes D, Levy K, Knee J, Handzel T, Hill VR (2015). Ascaris and Escherichia coli Inactivation in an ecological sanitation system in Port-au-Prince, Haiti. PLoS ONE..

[43] Phiri K, Whitty CJ, Graham SM, Ssembatya-Lule G (2000). Urban/rural differences in prevalence and risk factors for intestinal helminth infection in southern Malawi. Ann Trop Med Parasitol.

[44] Slum Dwellers International. Building EcoSan toilets in Blantyre, Malawi. 2015. http://sdinet.org/2015/12/11297/. Accessed 12 Feb 2016.

[45] Ahmed S, Creanga AA, Gillespie DG, Tsui AO (2010). Economic Status, education and empowerment: implications for maternal health service utilization in developing countries. PLoS ONE.

[46] Jencks C. Who gets ahead? The determinants of economic success in America. 1979 Aug 25. http://eric.ed.gov/?id=ED183100. Accessed 12 Feb 2016.

[47] Rogers EM (1983). Diffusion of innovations.

